# Application of causal inference methods in individual-participant data meta-analyses in medicine: addressing data handling and reporting gaps with new proposed reporting guidelines

**DOI:** 10.1186/s12874-024-02210-9

**Published:** 2024-04-19

**Authors:** Heather Hufstedler, Nicole Mauer, Edmund Yeboah, Sinclair Carr, Sabahat Rahman, Alexander M. Danzer, Thomas P. A. Debray, Valentijn M.T. de Jong, Harlan Campbell, Paul Gustafson, Lauren Maxwell, Thomas Jaenisch, Ellicott C. Matthay, Till Bärnighausen

**Affiliations:** 1 Heidelberg Institute of Global Health, Faculty of Medicine and University Hospital, Heidelberg University, Heidelberg, Germany; 2 Center for Interdisciplinary Addiction Research, University Medical Center Hamburg-Eppendorf, Hamburg, Germany; 3 University of Massachusetts Medical School, University of Massachusetts, Worcester, USA MA; 4 KU Eichstätt-Ingolstadt, Ingolstadt School of Management and Economics (WFI), Ingolstadt, Germany; 5 IZA, Bonn, Germany; 6 CESifo, Munich, Germany; 7 Julius Center for Health Sciences and Primary Care, University Medical Center Utrecht, Utrecht University, Utrecht, Netherlands; 8 Cochrane Netherlands, University Medical Center Utrecht, Utrecht University, Utrecht, Netherlands; 9 Smart Data Analysis and Statistics B.V, Utrecht, The Netherlands; 10 Department of Statistics, University of British Columbia, Vancouver, Canada BC; 11 Center for Global Health, Colorado School of Public Health, Aurora, USA CO; 12 Department of Epidemiology, Colorado School of Public Health, Aurora, USA; 13 Department of Population Health, New York University Grossman School of Medicine, New York City, USA NY; 14 Harvard T H Chan School of Public Health, Harvard University, Boston, USA MA; 15 Department of Epidemiology, Harvard T.H. Chan School of Public Health, Harvard University, Boston, MA USA

**Keywords:** Causal inference, Individual participant data, Meta-analysis, Longitudinal observational data, Pooling, Cohort studies

## Abstract

Observational data provide invaluable real-world information in medicine, but certain methodological considerations are required to derive causal estimates. In this systematic review, we evaluated the methodology and reporting quality of individual-level patient data meta-analyses (IPD-MAs) conducted with non-randomized exposures, published in 2009, 2014, and 2019 that sought to estimate a causal relationship in medicine. We screened over 16,000 titles and abstracts, reviewed 45 full-text articles out of the 167 deemed potentially eligible, and included 29 into the analysis. Unfortunately, we found that causal methodologies were rarely implemented, and reporting was generally poor across studies. Specifically, only three of the 29 articles used quasi-experimental methods, and no study used G-methods to adjust for time-varying confounding. To address these issues, we propose stronger collaborations between physicians and methodologists to ensure that causal methodologies are properly implemented in IPD-MAs. In addition, we put forward a suggested checklist of reporting guidelines for IPD-MAs that utilize causal methods. This checklist could improve reporting thereby potentially enhancing the quality and trustworthiness of IPD-MAs, which can be considered one of the most valuable sources of evidence for health policy.

**Supplementary Information:**

The online version contains supplementary material available at 10.1186/s12874-024-02210-9.

## Introduction

Randomized controlled trials (RCTs) are often considered the gold standard for establishing causal relationships. However, they may not always be feasible or ethical, particularly when dealing with exposures that cannot be randomized (e.g., cancer, obesity) or other exposures that would present ethical issues (e.g., Ebola Virus, smoking). Observational study designs are more often less resource-intensive and have the ability to evaluate the effects of a wider range of exposures than RCTs. This can allow for a larger number of individuals to be studied over a longer period of time.

In population health or global health science, where the goal is to make population-level inferences, meta-analyzing results from multiple studies can be an efficient and cost-effective way to increase statistical power and explore heterogeneity of single study findings across different sites, settings or populations [1]. There are two ways to conduct a meta-analysis (MA): pooling estimates (the traditional approach, also known as an aggregate data MA, which we do not review in this paper) and pooling individual-level patient data (IPD) to conduct a combined analysis. IPD-MAs are widely considered the gold standard in evidence-based medicine, as they often provide more precise and reliable estimates than MAs of aggregate data.

Aggregate data MAs may provide similar estimates to IPD-MAs in some settings [2, 3], but they are more prone to reporting bias [4], publication bias [5], or low statistical power [6]. IPD-MAs offer several other benefits, including the ability to adjust for confounders across studies, thereby minimizing the impact of between-study heterogeneity and reducing ecological bias [7, 8]. Moreover, data quality can be evaluated (e.g., study design features including randomization or follow-up) [9], IPD-MAs may have greater power to conduct subgroup analyses [9, 10], and they provide an opportunity to test assumptions of models and include unreported data [10]. However, IPD-MAs also present key challenges, such as accessing relevant data sources, data harmonization, and handling missing data in each study.

To address threats to internal validity present in observational studies when estimating causal effects in health science, the most commonly used approach is to include potential confounders as covariates in a standard regression-based adjustment (RBA) analysis, which we define as the investigation of a statistical relationship between a dependent and one (or more) explanatory variables. However, RBAs may not adequately control for measured confounding in the presence of time-varying confounders affected by prior treatment [11]. Analytical tools such as the G-methods (Marginal Structural Models [MSM], G-formula, and structural nested models) were developed to address these issues [12, 13]. Unmeasured confounding is another threat to internal validity in observational studies. In certain circumstances, data will allow for methods such as difference-in-differences [14], interrupted time series [15, 16], regression discontinuity design [17], and instrumental variables analysis [18, 19], methods which can circumvent unmeasured confounding. However, in other cases, including a sensitivity analysis for unmeasured confounders might be the only possible approach [20]. The strength of the inference relies not only on the method selected but also on the rigor with which the required assumptions are evaluated and tested.

Several reviews have shown that causal methods, which employ statistical techniques beyond above-mentioned standard RBA analyses, are implemented in single observational studies in medicine [21, 22]. However, a recent review [23] revealed that causal methods are rarely applied to IPD-MAs with infectious disease data. The objective of this systematic review is to expand on the previous review [23], and investigate the rigor in the implementation and reporting of causal methods in pooled longitudinal IPD studies in medicine.

## Methods

### Search strategy

The search strategy for this systematic review was developed by four researchers (HH, LM, EM, SR) and was reviewed and edited by information scientists from University Hospital Heidelberg (UKHD), University of California San Francisco (UCSF), and Harvard University. Similar to a previous review on infectious diseases [23], we chose not to include names of methods we considered “causal” but instead, allowed for methods not considered “causal”, such as standard RBAs, to be reviewed to prevent bias in the results. The search strategy was tailored to four large platforms so as to include non-medical disciplines (EBSCO [PsycINFO, Academic Search Complete, Business Source Premier, CINAHL, EconLit], EMBASE, PubMed and Web of Science). Details of the search strategy can be found in Supplementary Material 1.

Prior to initiating the systematic review, a protocol was registered with PROSPERO (CRD42020143148). Studies were included if they (1) posed a clear causal question related to the effect of an exposure on a health outcome, (2) estimated an effect size directly related to the causal question, and (3) pooled longitudinal individual-level data from more than one study or cohort. If a study pooled longitudinal data from RCTs, it was eligible for inclusion as long as not all of the exposure variables of interest were randomized (i.e., randomized exposures included in the pooled study must have been combined with non-randomized exposure variables). Furthermore, eligible studies had to be published (4) in the English language, (5) in peer-reviewed journals (accessible in full-text through open access, university licenses or project collaborators), and (6) in the years 2009, 2014, or 2019 (according to the electronic publication date). Due to resource constraints, the review was limited to publications at these three time-points, which were five years apart. Additional details about the study selection process are provided elsewhere [24].

### Study selection process

Search results were deduplicated in Endnote [25], version X9. Titles, abstracts and full-texts articles were screened in COVIDENCE systematic review software [26] by two reviewers each (SC, HH, NM, EY) using the double-blind tool. Discrepancies were resolved by consensus. This review originally sought to investigate the rigor of causal methods implementation and reporting across academic disciplines. However, as there were too few studies meeting inclusion criteria in non-medical fields (5 of 210 articles after title-abstract screening), making rigorous comparisons of implementation and reporting across disciplines was not feasible. Therefore, studies from fields other than medicine were excluded, and the focus of this review therefore shifted from ‘across disciplines’ to ‘within medicine’. As the search returned more studies than could feasibly be reviewed, we selected a random sample of 20% (*n* = 24) of eligible records using a stratified random sampling approach based on the year of publication. Randomly selected articles that did not meet the inclusion criteria (*n* = 11) were replaced by another random sample (*n* = 21) taken from the remaining pool of eligible articles.

### Data collection process

Data from each article were extracted using a predefined, peer-reviewed extraction form that consisted of over 70 points and was based on the PRISMA-IPD reporting guidelines [27] related to the pooling of studies (see Supplementary Material 2 Data Extraction Form). The extraction form also contained many reporting items related to causal methods implementation, as published in reporting guidelines for mediation analysis [28] and mendelian randomization [29]. Extracted data were cross-checked by at least two reviewers (SC, HH, NM, EY), and conflicts resolved by discussion or a tie-breaker (AD, VDJ). For each study, details such as (i) study design, (ii) statistical methods implemented, (iii) reporting of methods, and (iv) evaluation of assumptions were extracted.

### Data analysis

To measure the quality of reporting across studies, we developed and applied a scoring system that consisted of the following domains: data harmonization, accounting for missing data, causal methods, data pooling, and confounder control. Each domain included specific criteria related to the quality of reporting within that domain and was weighted equally. If a specific item from the data extraction list was not mentioned in the study documentation, 0 points were awarded. If the item was alluded to but not clearly addressed, 0.5 points were awarded. If the item was clearly addressed, 1 point was awarded. Findings of this systematic review are reported following the 2020 PRISMA Statement [30].

## Results

### Study selection

**Fig. 1:**
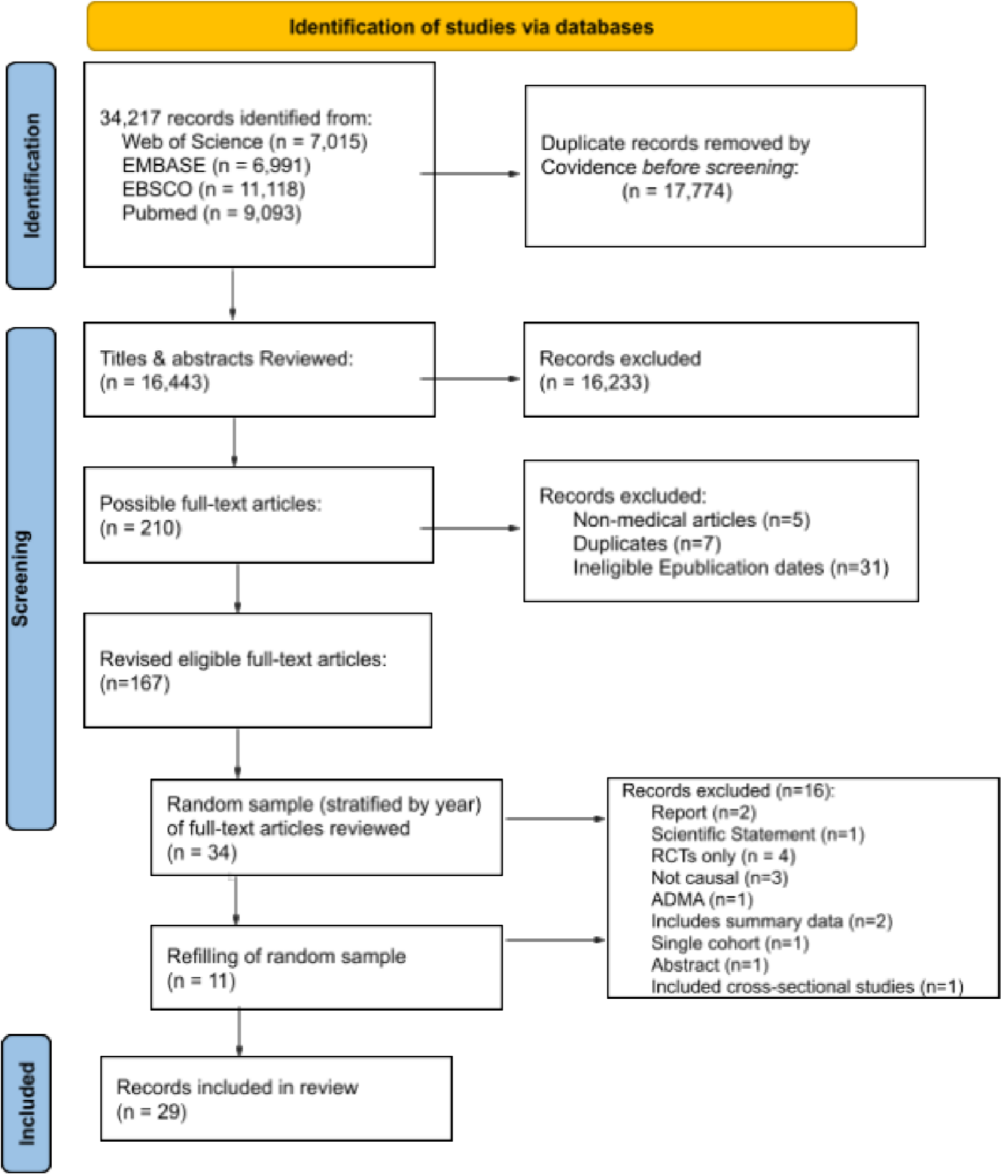
PRISMA flow diagram. Note. ADMA = aggregate data meta-analyses, ePUB = electronic publication, RCTs = randomized controlled trials

The search strategy yielded 16,443 unique articles. Of the 210 articles which were eligible at the initial title-abstract phase, seven duplicates and 31 articles with e-publication dates other than eligible years were excluded, as well as the five non-medical articles (explained in sections 2.2 Study Selection process), resulting in 167 eligible medical articles for full-text review (2009, *n* = 23; 2014, *n* = 44; 2019, *n* = 100), general medicine (32), neoplasms (24), vascular disease (23), internal medicine (15), public health (15), nutritional sciences (9), neurology (7), endocrinology (7), surgery, other specialty (6), environmental health (5), psychiatry (5), communicable diseases (4), drugs therapy (2), genetics (2), geriatrics (2), pregnancy (2), therapeutics (2), allergy and immunology (1), complementary therapies (1), critical care (1), dentistry (1), and metabolism (1). See Supplementary Material 3 Full Article List for information on all 167 eligible articles as well as the 45 articles which were reviewed in both of the random samples. Of the 45 articles reviewed, 29 articles [31–58] were included in the final analysis (see Fig. 1. PRISMA flow diagram).

### Study characteristics

Of the 29 IPD-MAs included in the final analysis, two were published in 2009, 10 in 2014, and 17 in 2019. The included IPD-MAs pooling data from cohort studies, RCTs, and case-control studies. The number of studies pooled in each IPD-MA ranged from 2 to 37, with an average of 12 studies. The pooled sample sizes ranged from 156 to 284,345 individuals. See the Supplementary Material 4. Details of Included Studies for more information.

### Reporting results

**Fig. 2:**
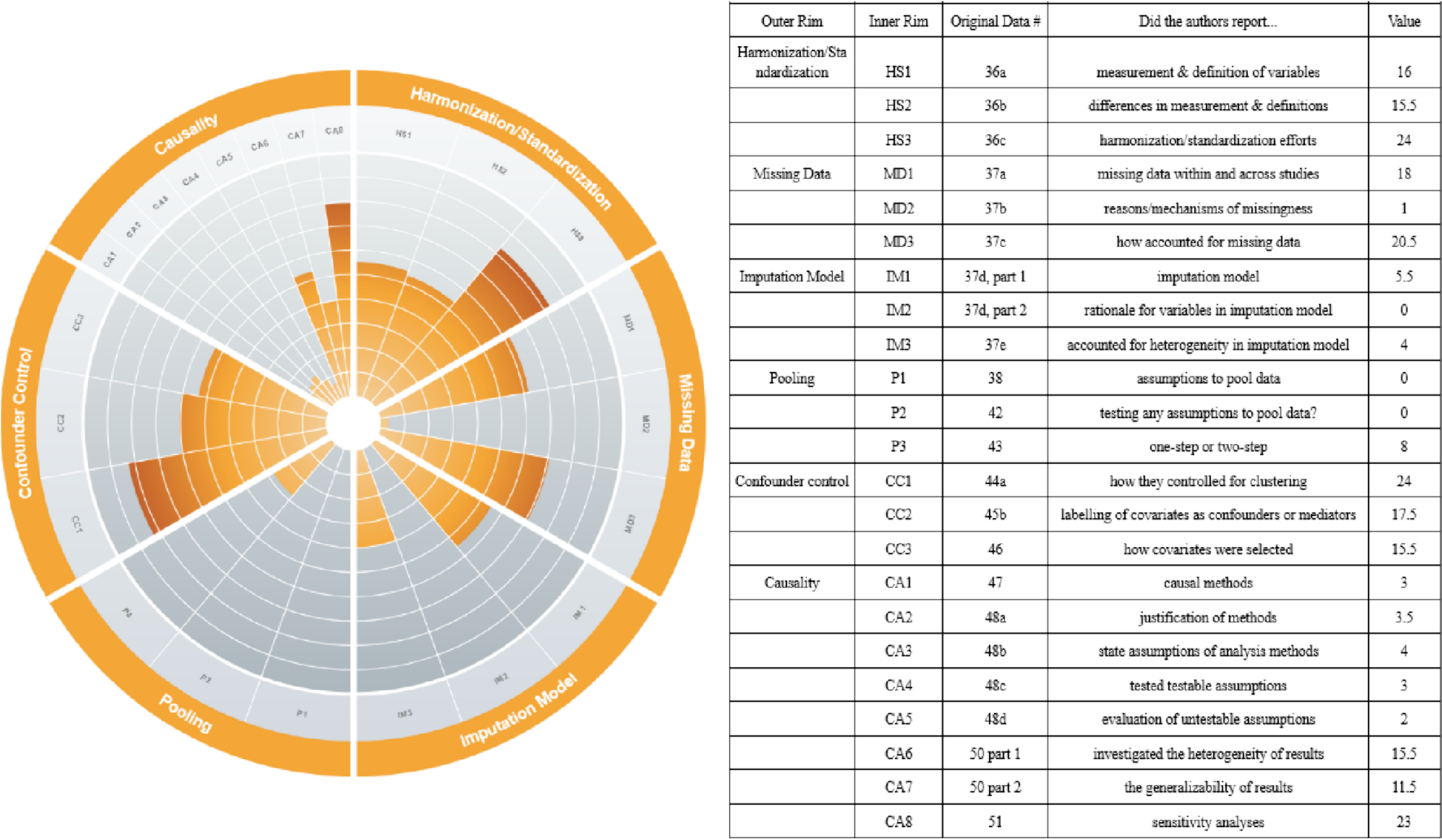
Bowman [62]. Summary results for data items that received points

Results of the data extraction are presented below. Please see Table 1 Results in tabular form and Fig. 2 Summary results for data items that received points for more details.

**Table 1:** Results in tabular form

Scoring domain	Criteria	Studies receiving 1 full point (n)	Studies receiving 0.5 points (n)	Studies receiving 0 points (n)	Total Number of Studies
Data harmonization	measurement & definition of variables	11 (37.9%)	10 (34.5%)	8 (27.6%)	*N* = 29
	differences in measurement & definitions	10 (34.5%)	11 (37.9%)	8 (27.6%)	*N* = 29
	harmonization/standardization efforts	20 (68.9%)	8 (27.6%)	1 (3.4%)	*N* = 29
Accounting for missing data	missing data within and across studies	11 (37.9%)	14 (48.3%)	4 (13.8%)	*N* = 29
	reasons/mechanisms of missingness	1 (3.4%)	0 (0%)	28 (96.6%)	*N* = 29
	how accounted for missing data	19 (65.5%)	3 (10.3%)	7 (24.1%)	*N* = 29
Imputation Model	Variables included in imputation model	5 (50%)	1 (10%)	4 (40%)	*N* = 10
	rationale for variables in imputation model	0 (0%)	0 (0%)	10 (100%)	*N* = 10
	accounted for heterogeneity in imputation model	4 (40%)	0 (0%)	6 (60%)	*N* = 10
Pooling	assumptions to pool data	0 (0%)	0 (0%)	29 (100%)	*N* = 29
	testing any assumptions to pool data?	0 (0%)	0 (0%)	29 (100%)	*N* = 29
	one-step or two-step	8 (27.6%)	0 (0%)	21 (72.4%)	*N* = 29
Causality	causal methods	3 (10.3%) ^a^	0 (0%)	26 (89.7%)	*N* = 29
	justification of methods	2 (6.9%)	3 (10.3%)	24 (82.8%)	*N* = 29
	state assumptions of analysis methods	4 (13.8%)	0 (0%)	25 (86.2%)	*N* = 29
	tested testable assumptions	3 (10.3%)	0 (0%)	26 (89.7%)	*N* = 29
	evaluation of untestable assumptions	2 (6.9%)	0 (0%)	27 (93.1%)	*N* = 29
	investigated the heterogeneity of results	13 (44.8%)	5 (17.2%)	11 (37.9%)	*N* = 29
	the generalizability of results	10 (34.5%)	3 (10.3%)	16 (55.2%)	*N* = 29
	Sensitivity analyses	23 (79.3%)	0 (0%)	6 (20.7%)	*N* = 29
Confounder Control	how they controlled for clustering	24 (82.8%)	0 (0%)	5 (17.2%)	*N* = 29
	labelling of covariates as confounders or mediators	15 (51.7%)	5 (17.2%)	9 (31%)	*N* = 29
	how covariates were selected	15 (51.7%)	1 (3.4%)	13 (44.8%)	*N* = 29

^a^ One used mediation analysis and two used propensity score analysis

Data points extracted without point assignment

Use of Weighting0 studies implemented weighting as part of the causal analysisReporting of results OR (16); HR(13); RR(1) *N* = 29

### Data harmonization

IPD-MAs were awarded 17 points of 29 possible points for describing the definitions and measurements of the variables collected from the individual cohorts. For describing the differences in definitions and measurements among the variables pooled, IPD-MAs were awarded 15.5 of 29 possible points, with 11 IPD-MAs providing insufficient detail, earning them a half-point each. IPD-MAs received 24 of 29 possible points for describing their efforts to manage the differences in variable definitions and measurements from individual cohorts, such as through harmonization or standardization.

### Accounting for missing data

18 of 29 possible points were awarded to IPD-MAs for describing the presence of missing data within and across studies (e.g. missing at random (MAR), missing not at random (MNAR), missing completely at random (MCAR)). Two IPD-MAs reported that the data was sporadically missing (i.e., data is partly missing in variables in one or more individual studies), and none of the IPD-MAs clearly labelled data as systematically missing (i.e., variables are entirely missing in one or more individual studies [59]). Twenty-one IPD-MAs were unclear about the type of missingness. One IPD-MA was awarded one point for a full description of why the data were missing, and 20.5 of 29 possible points were given to IPD-MAs for describing how the authors accounted for missing data.

### Imputation model

Of the 10 IPD-MAs using imputation methods to account for missing data, five-and-a-half of 10 possible points were given for listing the variables included in the imputation model. None of the IPD-MAs were awarded points for justifying the choice of variables in the imputation model, and four of the 10 IPD-MAs received full points for describing efforts to account for potential heterogeneity between studies in the imputation models.

### Data pooling

No points were awarded for discussing assumptions required for the methods they have used to pool the data, and no IPD-MA received points for describing the testing or evaluating of assumptions for the pooling method. Eight of the 29 IPD-MAs received full-points for clearly stating whether they implemented a one-step (*n* = 4) or two-step (*n* = 4) meta-analysis [60, 61].

### Causal methods

Out of the 29 IPD-MAs, three used causal methods (one used mediation analysis; two used propensity scores), while the remaining 26 used standard regression-based analyses, including Cox proportional hazards regression, logistic regression, and linear regression. While most studies reported drawing data from longitudinal studies, exact time-points for variables included in the analyses was not clearly reported across IPD-MAs. Three-and-a-half of 29 possible points were awarded for justifying the choice of method used for causal inference or other statistical methods used. Four points out of 29 were awarded for explicitly stating assumptions required for the causal inference or statistical modelling approach selected. Three points were awarded for reporting the testing at least one of the testable assumptions, all of which were proportional hazards assumption. Two IPD-MAs discussed the evaluation of untestable assumptions (e.g., no unmeasured confounding) and thus received one point each. No IPD-MA implemented weighting in their causal analyses. 15.5 points were awarded to IPD-MAs for reporting that they investigated the potential for heterogeneity in the results. Eleven-and-a-half of 29 possible points were awarded to IPD-MAs for discussing the possible impact of any heterogeneity on the generalizability of the results. Twenty-three points were awarded for the reporting of sensitivity analyses.

### Confounder control

Twenty-four points out of 29 were awarded for reporting the method(s) used to account for clustering/heterogeneity at the cohort level. These methods included stratification (*n* = 12), random effects (*n* = 9), interaction terms (*n* = 2), confounder adjustment (*n* = 2), and fixed-effects (*n* = 1). Seventeen-and-one-half points were awarded to IPD-MAs for indicating how the covariates were conceptualized (e.g., confounders, mediators), and 15.5 points were awarded for describing how they selected their covariates— e.g., two IPD-MAs [43, 55] reviewed the literature; one [36] used statistical testing procedures.

### Effect estimates

Most IPD-MAs did not clearly report how they accounted for (potential) heterogeneity. Due to these ambiguities, we used a rough categorization based on our assumptions about the authors’ intentions: five IPD-MAs took strata-specific estimates; three IPD-MAs excluded specific patients or data points responsible for baseline heterogeneity; two IPD-MAs suggest the use of data standardization or harmonization was used to account for this; one IPD-MA reported that they found no baseline heterogeneity; and the remaining IPD-MAs (*n* = 17) only reported adjusting for variables. It was unclear for any of the studies which effect—marginal or conditional— was estimated. However, we inferred that 10 IPD-MAs estimated a conditional effect, one IPD-MA estimated a marginal effect, and four IPD-MAs estimated both. For the remaining IPD-MAs, no precise statement can be made.

## Discussion

This systematic review evaluates the use of causal methods in IPD-MAs in medicine. Specifically, we investigated the implementation and reporting of methods to address causal questions and the critical role of data handling and reporting in this context. Overall, we found that the use of standard regression methods was the most common approach, and note the lack of utilization of other causal methods tailored to address time-varying and unmeasured confounding. While sensitivity analyses can be used to alleviate concerns with unmeasured confounding [20], these were not reported to have been used. We also observed major gaps in the reporting of the methodology used in pooled longitudinal, observational studies, including issues related to harmonization, missing data, and data standardization—all crucial to the implementation of causal methods. Table 2 provides guidance as to the various technical aspects that researchers engaging in IPD-MAs should consider for robust and reliable results.

### Pooled studies

Our results suggest an increase in the number of pooled longitudinal observational studies within the medical field between 2009 and 2019. This upward trend mirrors the findings of an earlier review [7], and may reflects a greater understanding of the importance of IPD-MAs [63], improved digitization of records and data sharing efforts, and/or improved statistical software (both for conducting IPD-MAs as well as causal methodologies).

### Causal methods

Although all included IPD-MAs were considered to have causal intent, all but three used standard RBA analyses. This finding is consistent with other reviews suggesting underutilization of causal methods in medicine or medical subfields [21, 23, 64]. This may be due to the high variability in data elements and study designs, which can pose challenges in applying certain methods for pooled data as compared to analyzing a single dataset. Alternatively, it could reflect a general lack of knowledge about or understanding of how to apply these methods to pooled studies. To address these issues, investigators may want to review (introductory) articles on causal methods [65–67]. We want to point out that no one method is universally better suited for one scientific field over another. Rather, each of these causal methods entail tradeoffs [68], and the choice of method should be determined by the research question at hand and the data available. In the context of causal analysis, researchers often rely on tools like Directed Acyclic Graphs (DAGs) to map the (assumed) relationships between factors, allowing them to identify potential confounders and mediators, e.g., and understand what variables need to be controlled or adjusted for [69]. This approach can provide valuable insights into the causal relationships under investigation and enhance the validity of results.

**Table 2:** Technical considerations required by IPD-MAs

Technical considerations	Descriptions
Harmonization	Harmonization efforts often require intensive feedback from stakeholders and can therefore take an enormous amount of time—one study reported that their harmonization took nearly two years [70], and a report by Cochrane Group members suggested that researchers should not expect their first publication before 3 years [9].
Missing Data	What to do with missing data becomes more complex as studies are combined into one meta-analysis as the reasons for missingness (varying protocols for measurement; some studies may not capture the variable of interest; or, if studies all capture the variable of interest, the reason for its missingness may also vary across studies). The choice methods to account for these differences, then, require additional consideration, e.g., omission, imputation. And, if the choice for imputation is made, thoughtful decisions about imputation models and the order in which to conduct the next steps (one-step, two-step, Rubin’s rules) are required.
Pooling	There are trade-offs with both methods [71]: one-step meta-analysis methods require advanced statistical know-how, and two-step methods, though employing more widely-known methods (e.g. random-effects or inverse-variance fixed-effects), are a more arduous endeavor. Two-step methods were the more dominant across all medicinal fields in the past, but the one-step methods have been increasing some medical fields as knowledge of and software to assist have improved [72]. In fields like epidemiology, one-step methods have been said to be the dominant choice due to adjustment for covariates other than treatment.
Causal Methods	As this review showed, authors of studies included in this review focused their reporting on confounding control. However, there are additional forms of bias which can be present in longitudinal studies, whether purely observational or pooled with RCTs, such as time-varying confounding. Causal methods can be extremely beneficial to remove some forms of bias, e.g., unmeasured confounding, time-varying confounding. Some of these methods are common in the disciplines where they were created, e.g., Regression Discontinuity in Economics, but have been recently increasing in medicine. Implementing these novel methods across multiple studies with different study designs may require additional time and resources initially but may yield powerful results for the field of global health and population medicine.

Several previous reviews have identified discrepancies in causal effects between RCTs and observational studies with the same exposures and outcomes [73]. As researchers in academia and industry are increasingly interested in the use of real-world evidence for regulatory decision making, IPD-MAs and other approaches to the pooled analyses of multiple longitudinal studies should consider applying causal methods (e.g., G methods with IV approaches) to account for time-varying confounding and unmeasured confounding.

### Reporting guidelines

While many IPD-MAs included in this systematic review reported variable definitions, measurement methods, and efforts to harmonize data, they would benefit from reporting additional details like, e.g., between-study differences in measurement methods and variable definitions, which were rarely discussed and may affect the validity and interpretation of causal effect estimates.

When following best practices for harmonization [74], authors should consider reporting their detailed efforts in appendices, where there is ample space. However, we found that descriptions of data standardization were low, potentially due to a lack of lack of understanding of data standard availability, usability, or lack of feasibility of implementation based on specific needs of epidemiological studies.

Our review also revealed that few of the included IPD-MAs described the type of missing data present; either *sporadically* missing values, when these data are missing on observations within a particular study, or *systematically* missing, when variables are not defined consistently across studies and therefore missing entirely from specific studies [59]. Many studies simply omitted participants with missing data, using complete cases, a common practice in medicine. Although multiple imputation is generally recommended to account for missing data, the implementation becomes problematic when variable definitions or measurement methods differ across studies. For this reason, several multi-level imputation methods have been proposed that are better capable of preserving between-study heterogeneity and uncertainty when imputing missing data in IPD-MA and other types of pooled cohort studies [75].

The strength of causal inferences that can be made from any approach rely on the rigor with which assumptions were tested (if testable) or evaluated (if untestable). Although there are many forms of bias that can adversely influence the results of a study (e.g., reverse causation, measurement error), authors reported almost entirely on confounding bias. We would recommend that the authors explicitly report other forms of bias that they considered in their analysis, both to make the interpretability of their results transparent and to inform future studies on similar research questions.

There are reporting guidelines which exist in JAMA for mediation analyses [28] and mendelian randomization analyses [29], as well as reviews of and suggested reporting checklists for Instrumental Variable analyses [76, 77] but all of these publications appear to be intended for use in single studies. In addition, despite reporting guidelines existing for IPD-MAs [27], researchers have previously reported lower-than-desired reporting patterns from authors of IPD-MAs with regards to their statistical methods [23, 72]. There are currently no reporting guidelines for IPD-MAs which employ causal methods. We, therefore, propose that reporting guidelines for pooled studies employing causal methodologies be developed (see Supplementary Material 5 Proposed Reporting Guidelines Checklist for IPD-MAs implementing Causal Methods), based on the aforementioned published reporting guidelines (see Supplementary Material 6 Reporting Guidelines Comparison). We would appreciate any feedback to the proposed checklist.

### Strength and limitations

Strengths of our systematic review include the search strategy, which was built on other systematic reviews of non-randomized exposures and reviews of similar methods and was also built in consultation with three experienced librarian scientists, and with input from colleagues in other fields regarding synonyms that could be used in other disciplines. The search strategy was also implemented in non-medical platforms to ensure potential identification of non-medical articles. The strategy also did not employ specific names, or variations of or acronyms of the names of the methods, so as not to bias our results by including only methods which we considered “causal”. Another strength is the sheer number of articles that we screened and data points that we extracted—nearly four and six times the number of titles and abstracts screened by similar reviews [21, 22], and far exceeded the extraction numbers of those same reviews (five and 24 items to our 70 + items).

Weaknesses of our systematic review include the scarcity of articles found from disciplines other than medicine. This low number could be because health outcomes from pooled data sets are not often being investigated in disciplines other than medicine, but we must also consider the possibility that this small number we found is related to the search strategy, despite the rigor with which it was built. We also recognize that the global generalizability of our results is limited due to language and year restrictions. We must also consider that, although we appeared to reach theoretical saturation, our findings may be limited by the fact we were only able to review roughly 27% of potentially-eligible full-text articles. Another limitation is that the review was limited to the *reporting* of the use of causal methods in IPD-MAs with in medicine which may not reflect what was actually *done*. Further, it is possible that we included some studies that did not (primarily) aim to infer a causal relationship as the study aims were not always entirely clear. We attempted to counter subjectivity with blind assessment by at least two reviewers per study, rounds of discussion between reviewers in case of disagreement, and consultation with additional scientists from four universities across four countries.

## Conclusions

To encourage better reporting and implementation of causal methods in future pooled longitudinal IPD studies, we propose the following approaches. First, we suggest that authors always clearly describe their methods. The domain criteria evaluated in this study can serve as a basis for developing or building on existing reporting standards. Although most medical journals set a predefined word limit for publications, the appendix, which usually has no word limit, is a simple way to include an in-depth description and justification of each aspect of the methodological approach. Second, the research community could publish accessible “how-to” documents that apply causal inference methods to pooled IPD studies and are accompanied by open-source data and code to ensure that investigators can better apply these methods to studies that pool longitudinal, observational data. This will lower the barrier to engaging with appropriate and potentially unfamiliar methods and could ultimately increase application of these methods in the broader research contexts, as well as inform health policy and decision making.

### Supplementary Information

**Supplementary Material 1.**

**Supplementary Material 2.**

**Supplementary Material 3.**

**Supplementary Material 4.**

**Supplementary Material 5.**

**Supplementary Material 6.**

## Data Availability

Data and materials can be found in the Supplementary Materials 1, 2, 3, 4, 5 and 6.

## Abbreviations

RCTs: Randomized controlled trials
IPD-MA: Individual-level patient data meta-analysis
RBA: Regression-based adjustment
