# Supplementary material for: Application of causal inference methods in individual-participant data meta-analyses in medicine: addressing data handling and reporting gaps with new proposed reporting guidelines
Source: BMC Med Res Methodol. 2024 Apr 19;24:91. doi: 10.1186/s12874-024-02210-9 (PMC11027270; doi:10.1186/s12874-024-02210-9)
Supplement: Supplementary file 1 — Supplementary Material 1. [file 12874_2024_2210_MOESM1_ESM.docx]

Supplementary Material 1. Search strategy

1. Web of Science
2. Embase
3. EBSCO (PsycINFO, Academic Search Complete, Business Source Premier, CINAHL, EconLit with Full Text)
4. Pubmed
5. Web of Science- Core

| **#** | **Searches** |
| --- | --- |
| 1 | TS=("individual patient data" OR "individual participant data" OR “individual-patient-data” OR “individual-participant-data” OR “individual-participant” OR “individual-patient” OR “participant data” OR “patient data” OR “individual-level") |
| 2 | TS=((“cohort” OR “longitudinal” OR “observational”) NEAR/2 (pool* OR harmoniz* OR harmonis*)) |
| 3 | #1 OR #2 |
| 4 | TS=("animal model" OR animal*) |
| 5 | #3 NOT #4 |
| 6 | TI=("single center" OR "single centre" OR "single-centre" OR "single-center" OR “multi center” OR “multi centre” OR “multi-centre” OR “multi-center” OR “multicenter” OR “multicentre” OR “multi-site” OR “predict*” OR “cross-sectional” OR “prognos*” OR “protocol” OR “erratum” or “correction” or “author correction”) |
| 7 | AB= ("single center" OR "single centre" OR "single-centre" OR "single-center" OR “multi center” OR “multi centre” OR “multi-centre” OR “multi-center” OR “multicenter” OR “multicentre” OR “multi-site” OR “predict*” OR “cross-sectional” OR “prognos*” OR “protocol” OR “erratum” or “correction” or “author correction”) |
| 8 | #6 OR #7 |
| 9 | #5 NOT #8 |
| 10 | TS=("RCT*" OR "Randomi?ed Controlled Trial*" OR "animal model" OR animal*) |
| 11 | #9 NOT #10 |
| 12 | #11 Refined by: [excluding] DOCUMENT TYPES: (PROCEEDINGS PAPER OR BOOK CHAPTER OR EDITORIAL MATERIAL OR LETTER OR REPRINT OR NEWS ITEM OR MEETING ABSTRACT OR CORRECTION OR BOOK REVIEW ) AND [excluding] |
| 13 | #12 in year 2019 |
| 14 | #12 in year 2014 |
| 15 | #12 in year 2009 |
| 16 | #13 OR #14 OR #15 |
| 17 | Limit 16 to English |

1. Embase

| **#** | **Searches** |
| --- | --- |
| 1 | "individual patient data":ti,ab OR "individual participant data":ti,ab OR “individual-patient-data”:ti,ab OR “individual-participant-data”:ti,ab OR “individual-participant”:ti,ab OR “individual-patient”:ti,ab OR “participant data”:ti,ab OR “patient data”:ti,ab OR “individual-level”:ti,ab |
| 2 | ((cohort OR longitudinal OR observational) NEAR/2 (pool* OR harmoniz* OR harmonis*)):ti,ab |
| 3 | ‘cohort studies’/de |
| 4 | 'longitudinal study'/de |
| 5 | pool*:ti OR cross*:ti OR harmoniz*:ti OR harmonis*:ti |
| 6 | #3 OR #4 |
| 7 | #5 AND #6 |
| 8 | #1 OR #2 OR #7 |
| 9 | 'single center':ti,ab OR 'single-center':ti,ab OR 'single centre':ti,ab OR 'single-centre':ti,ab OR 'multi center':ti,ab OR 'multi centre':ti,ab OR ‘multicenter’:ti,ab OR ‘multicentre’:ti,ab OR 'multi site':ti,ab OR ‘multisite’:ti,ab OR 'multiple sites':ti,ab OR 'cross sectional':ti,ab OR ‘transversal’:ti,ab OR ‘prognos*’:ti,ab OR ‘predict’:ti,ab OR 'randomized control*':ti,ab OR 'randomised control*':ti,ab OR 'randomised clinical':ti,ab OR 'randomized clinical':ti,ab OR 'randomized trial*':ti,ab OR 'randomised trial*':ti,ab OR ‘rct’:ti,ab OR ‘clinical trial*':ti,ab OR ‘protocol’:ti,ab OR ‘erratum’:ti,ab OR ‘correction’:ti,ab or ‘author correction’:ti,ab |
| 10 | #8 NOT #9 |
| 11 | #10 AND ‘randomized controlled trial’/de AND ‘clinical trial’/de |
| 12 | #10 NOT #11 |
| 13 | ([animals]/lim NOT [humans]/lim) |
| 14 | #12 NOT #13 |
| 15 | #14 AND 'conference abstract'/it |
| 16 | #14 NOT #15 |
| 17 | #16 AND [2009]/py |
| 18 | #16 AND [2014]/py |
| 19 | #16 AND [2019]/py |
| 20 | #17 OR #18 OR #19 |

1. EBSCO-- **Academic Search Complete, Business Source Premier, CINAHL, EconLit with Full Text, PyscINFO**

| **#** | **Searches** |
| --- | --- |
| 1 | AB ("individual patient data" OR "individual participant data" OR “individual-patient-data” OR “individual-participant-data” OR “individual-participant” OR “individual-patient” OR “participant data” OR “patient data” OR “individual-level”) |
| 2 | AB ((“cohort”) OR (“longitudinal”) OR (“Observational”)) N2 ((pool*) OR (harmoniz*) OR (harmonis*)) |
| 3 | S1 OR S2 |
| 4 | SU (animal experimentation or animal testing or animal research) |
| 5 | S3 NOT S4 |
| 6 | TI ("single center" OR "single centre" OR "single-centre" OR "single-center" OR “multi center” OR “multi centre” OR “multi-centre” OR “multi-center” OR “multicenter” OR “multicentre” OR “multi-site” OR “cross-sectional” OR “predict*” OR “prognos*” OR “protocol” OR “erratum” OR “correction” OR “author correction") |
| 7 | AB ("single center" OR "single centre" OR "single-centre" OR "single-center" OR “multi center” OR “multi centre” OR “multi-centre” OR “multi-center” OR “multicenter” OR “multicentre” OR “multi-site” OR “cross-sectional” OR “predict*” OR “prognos*” OR “protocol” OR “erratum” OR “correction” OR “author correction") |
| 8 | S6 OR S7 |
| 9 | S5 NOT S8 |
| 10 | SU (randomized controlled trials OR rct OR randomised control trial OR randomized control trial OR randomized clinical trial OR randomised clinical trial OR randomized controlled study OR animal experimentation or animal testing or animal research) |
| 11 | S9 NOT S10 |
| 12 | SO editorial or opinion or commentary |
| 13 | S11 NOT S12 |
| 14 | Limit to Journals and Academic Journals |
| 15 | S14 limit to 2019 |
| 16 | S14 limit to 2014 |
| 17 | S14 limit to 2009 |
| 18 | S15 OR S16 OR S17 |
| 19 | Limit 17 to English |

1. Pubmed

| 1 | "individual-patient-data"[Title/Abstract] OR "individual-participant-data"[Title/Abstract] OR "individual-patient-data"[Title/Abstract] OR "individual-participant-data"[Title/Abstract] OR "individual-participant"[Title/Abstract] OR "individual-patient"[Title/Abstract] OR "participant data"[Title/Abstract] OR "patient data"[Title/Abstract] OR "individual-level"[Title/Abstract] |
| --- | --- |
| 2 | ("cohort"[Title/Abstract] OR "longitudinal"[Title/Abstract] OR "observational"[Title/Abstract]) AND (pool*[Title/Abstract] OR harmoniz*[Title/Abstract] OR harmonis*[Title/Abstract]) |
| 3 | #1 OR #2 |
| 4 | "animal experimentation"[MeSH Terms] OR "animal testing alternatives"[MeSH Terms] OR "animal experimentation"[MeSH Terms] |
| 5 | #3 NOT #4 |
| 6 | "single-center"[Title/Abstract] OR "single-centre"[Title/Abstract] OR "single-centre"[Title/Abstract] OR "single-center"[Title/Abstract] OR "multi-center"[Title/Abstract] OR "multi-centre"[Title/Abstract] OR "multi-centre"[Title/Abstract] OR "multi-center"[Title/Abstract] OR "multicenter"[Title/Abstract] OR "multicentre"[Title/Abstract] OR "multi-site"[Title/Abstract] OR "predict*"[Title/Abstract] OR "cross-sectional"[Title/Abstract] OR "prognos*"[Title/Abstract] OR "protocol"[Title/Abstract] OR "erratum"[Title/Abstract] OR "correction"[Title/Abstract] OR "author correction"[Title/Abstract] |
| 7 | #5 NOT #6 |
| 8 | "randomized controlled trials as topic"[MeSH Terms] OR "animal experimentation"[MeSH Terms] OR "animal testing alternatives"[MeSH Terms] OR "animal experimentation"[MeSH Terms] |
| 9 | #7 NOT #8 |
| 10 | "address"[Publication Type] OR "autobiography"[Publication Type] OR "bibliography"[Publication Type] OR "biography"[Publication Type] OR "book illustrations"[Publication Type] OR "webcast"[Publication Type] OR "case reports"[Publication Type] OR "clinical trial, veterinary"[Publication Type] OR "collected work"[Publication Type] OR "collected works"[Publication Type] OR "comment"[Publication Type] OR "consensus development conference"[Publication Type] OR "dataset"[Publication Type] OR "dictionary"[Publication Type] OR "directory"[Publication Type] OR "duplicate publication"[Publication Type] OR "editorial"[Publication Type] OR "electronic supplementary materials"[Publication Type] OR "ephemera"[Publication Type] OR "equivalence trial"[Publication Type] OR "evaluation studies"[Publication Type] OR "evaluation study"[Publication Type] OR "expression of concern"[Publication Type] OR "festschrift"[Publication Type] OR "interactive tutorial"[Publication Type] OR "interview"[Publication Type] OR "lecture"[Publication Type] OR "legal case"[Publication Type] OR "legislation"[Publication Type] OR "letter"[Publication Type] OR "news"[Publication Type] OR "newspaper article"[Publication Type] OR "observational study, veterinary"[Publication Type] OR "patient education handout"[Publication Type] OR "periodical index"[Publication Type] OR "personal narrative"[Publication Type] OR "pictorial work"[Publication Type] OR "portrait"[Publication Type] OR "published erratum"[Publication Type] OR "randomized controlled trial"[Publication Type] OR "randomized controlled trial, veterinary"[Publication Type] OR "retracted publication"[Publication Type] OR "retraction of publication"[Publication Type] OR "video-audio media"[Publication Type] OR "validation study"[Publication Type] |
| 11 | #9 NOT #10 |
| 12 | Limit #11 to 2009/01/01:2009/12/31[Date - Publication] OR 2014/01/01:2014/12/31[Date - Publication] OR 2019/01/01:2019/12/31[Date - Publication] |
| 13 | Limit 12 to English |
