# Supplementary material for: Application of causal inference methods in individual-participant data meta-analyses in medicine: addressing data handling and reporting gaps with new proposed reporting guidelines
Source: BMC Med Res Methodol. 2024 Apr 19;24:91. doi: 10.1186/s12874-024-02210-9 (PMC11027270; doi:10.1186/s12874-024-02210-9)
Supplement: Supplementary file 2 — Supplementary Material 2. [file 12874_2024_2210_MOESM2_ESM.docx]

Supplementary Material 2. Data Extraction Sheet

| Title of the paper/article/report |
| --- |
|  |
| DOI -- |
|  |
| Study ID (surname of first author and year first full report of study was published e.g. Smith 2001 |
|  |
| Report IDs of other reports of this study (e.g. duplicate publications, follow-up studies) |
|  |
| Notes |

1. General Information (Example entries)

| 1. Date form completed  (dd/mm/yyyy) |  |
| --- | --- |
| 2. Name/ID of person extracting data |  |
| 3. Title of article (title of paper/abstract/report that data are extracted from) |  |
| 4. First author |  |
| 5. Contact Details |  |
| 6. Publication type (e.g. full report, abstract, letter) |  |
| 7. Possible conflicts of interest (for study authors) |  |
| 8. Notes |  |

2. Eligibility to be included in this systematic review (no inclusion/exclusion criteria for study participants)

| Study Characteristics | Copy and paste related descriptions as stated in report/paper | Location in text (page and paragraph/figure/table) |
| --- | --- | --- |
| 8. Is the study published in either 2009, 2014, or 2019? (consider the electronic publication date) If not, exclude. |  |  |
| 9. Is the “parent” study  composed of ≥2 separate observational and longitudinal studies? If yes, include. If only single-site or multi-site, or single-cohort, exclude. If cross-sectional or repeated cross-sectional, exclude. If it includes randomized controlled trials (RCTs), go to the next question. |  |  |
| 10. Are any of the included studies RCTs? If yes, is the “parent” study using non-randomized variables from at least one RCT or observational cohort in the analysis? If yes, include. If not, exclude. |  |  |
| 11. Are study subjects human? If animals, human tissue samples, genetics, or similar, exclude. If studies include individual genetic data (e.g., single nucleotide polymorphism for Mendelian Randomization), in addition to observational data from humans in their analyses, include. |  |  |
| 12. Is it focused on health outcomes? If not (e.g. wages, salaries), exclude. If outcomes that are not a health outcome in principle (e.g., score on Mini-Mental State Test) are clearly declared as a proxy for an (underlying) health outcome, include. |  |  |
| 13. Does the paper include case studies? If yes, exclude. |  |  |
| 14. Is it a methods paper with the primary goal of describing, developing, or summarizing a statistical analytical method, with no original analysis of pooled observational, longitudinal data? If yes, exclude. If with an applied real-life example pooling individual participant data, include. |  |  |
| 15. Is the paper a protocol, review, commentary correction, editorial, erratum, or similar? If yes, exclude. |  |  |
| 16. Is the paper in English? If not, exclude. |  |  |
| 17. Is the data clearly pooled on an individual level? If not, exclude. If the language indicates that individual participant data were pooled and the analyses applied (e.g., Cox proportional hazards model) are only possible with individual participant data, include. |  |  |
| 18. Does the study attempt to establish a causal relationship? If 1) causal inference methods (as described in the protocol; e.g., regression  discontinuity design, instrumental variable approach,  G-methods) were used,  or 2) the language suggests a causal intent AND the regression-based analysis (e.g., Cox proportional hazards model) adjusted for a set of possible confounders, include. If the aim of the study is descriptive, predictive, or prognostic, exclude. If uncertain,  label as uncertain. |  |  |
| 19. Is an effect size estimated? |  |  |
| 20. Is the effect size directly related to the causal question? If the effect size does not correspond to the stated causal research question, exclude. |  |  |
| 21. Decision to include |  |  |
| 22. Notes |  |  |

DO NOT PROCEED IF STUDY IS EXCLUDED FROM REVIEW

3. Characteristics of individual datasets/studies included in the pooled analysis

|  | Copy and paste related descriptions as stated in report/paper | Location in text (page & paragraph/figure/table) |
| --- | --- | --- |
| Individual studies | | |
| 23. List number & types of individual studies or cohorts included in the pooled analysis (e.g. 3 cohort studies, 2 case-control studies, patient registries) |  |  |
| 24. Study populations of each individual study or cohort included in the pooled analysis |  |  |
| 25. Number of participants in each individual study or cohort included in the pooled analysis |  |  |
| 26. Recruitment period of each individual study or cohort included in the pooled analysis |  |  |
| 27. Location of data collection of each individual study or cohort included in the pooled analysis |  |  |
| Pooled cohort study | | |
| 28. In which journal was the pooled cohort (“parent”) study published? |  |  |
| 29. Discipline of “parent” study? (based on metrics developed in the protocol) |  |  |
| 30. Country of affiliation of primary author |  |  |
| 31. What exposures are studied in “parent” study? List which are randomized and which are non-randomized. |  |  |
| 32. What are the primary outcomes in the “parent” study? (e.g. myocardial infarction, hypertension, remission) |  |  |
| 33. Funding Source: Copy & paste the funding section of the manuscript here. |  |  |
| 34. Key conclusions of study authors (of pooled study data, not single studies) From abstract section. |  |  |
| 35. Notes |  |  |

4. Methods and reporting standards

|  | Copy and paste related descriptions as stated in report/paper | Location in text (page & paragraph/figure/table) |
| --- | --- | --- |
| 36a. Did the study describe how variables were measured and defined in each study? |  |  |
| 36b. Did the study describe any differences in measurement and definition of variables across studies? |  |  |
| 36c. If 36b is yes, did the study discuss how it dealt with differences in variable definitions and measurement methods? (standardization or harmonization) |  |  |
| 37a. Did the study describe the presence of missing data within and across studies? (e.g. presence of sporadically and systematically missing values) |  |  |
| 37b. Did the study describe possible reasons/mechanisms of missingness? |  |  |
| 37c. How did the study account for missing data within and across studies? Specifically, what method was used for the primary analysis? (e.g. omission of patients with missing values or multiple imputations) |  |  |
| 37d. In case imputation was used, do the authors discuss what variables were included in the imputation model and why? |  |  |
| 37e. In case of imputation, what efforts were made to account for potential heterogeneity between studies? (e.g. impute each study separately, or adopt multilevel imputation methods) |  |  |
| 38. Do the authors discuss any of the assumptions required for the analysis methods they have chosen to pool the data? If yes, which ones? Copy and paste relevant text describing the tests or reporting the results of those tests, if any, here. If not reported, write “not reported”. If unclear, write “unclear”. |  |  |
| 39. What estimation method was used for deriving the (pooled) causal effect? Specific for which parameter the estimation method is being used Multiple estimation methods can be used for different parameters (e.g. estimation of propensity model versus estimation of analysis model). |  |  |
| 40. Are authors estimating a marginal or conditional effect? (yes, no, unclear) |  |  |
| 41. Pooled analysis – What type of estimand is used? |  |  |
| 42. Do the authors report testing any of the assumptions required for the analysis methods they have chosen to pool the data? If yes, which ones? Copy and paste relevant text describing the tests or reporting the results of those tests, if any, here. If not reported, write “not reported”. If unclear, write “unclear”. |  |  |
| 43. Did the authors analyze each dataset separately and pooled the corresponding results? (or did they analyze all data directly using a so-called one-stage approach?)    If YES, please define what method was used to pool results across studies (e.g. random effects meta-analysis)    If NO, go to question 44a |  |  |
| 44a. List approach(es) to account for clustering/ heterogeneity at the cohort or pooled study level (whichever units are pooled across)  (note whether this is done to stratify within or across studies) |  |  |
| 44b. How did they adjust for (potential) heterogeneity in baseline risk, confounder effects, mediator effects, causal effects etc.? |  |  |
| 45a. Which covariates were adjusted for in the analysis? (e.g. by considering them as adjustment in regression, or as a variable of propensity score model, or as a matching variable) |  |  |
| 45b. Were they labeled as confounders or mediators of the causal relationships? If yes, list them. |  |  |
| 46. On what basis were the confounders selected? Studies may have restricted to a set of confounders because those were the most commonly measured variables across studies, or defined a list of confounders based on a directional acyclic graph (DAG) and imputed study level information for systematically missing confounders; or combined fully and partially adjusted studies in a multivariate approach. |  |  |
| 47. Which methods were used with the pooled data to make causal inferences? (e.g. interrupted time series with a control group; comparative study without concurrent controls; IV; Mendelian randomization; RD; interrupted time series, including DiD estimation; G-estimation; multiple regression adjusting for confounders; propensity score matching; inverse probability of treatment weighting) |  |  |
| 48a. Justification for method(s) used (e.g. “we selected a synthetic control approach because this method is well-suited to situations involving 1 intervention unit, and many controls and may better approximate counterfactual post-intervention outcomes than using any single control or an evenly weighted combination of controls” or “This approach is advantageous, because characteristics of each region, other than the occurrence of the treatment, are unlikely to change appreciably over so short a time period. Thus, each region serves as its own control, allowing us to control for other community-level characteristics that may also be associated with injuries.”) |  |  |
| 48b. Did the authors explicitly state the assumptions required for causal inference methods? If yes, which ones? (e.g. ignorability, positivity, stable unit treatment value, transitivity) Copy and paste relevant text, if any, here. If not reported, write “not reported”. If unclear, write “unclear”. |  |  |
| 48c. Do the authors report testing any of the testable assumptions required for the analysis methods they have chosen to deliver causal effects? If yes, which ones? Copy and paste relevant text describing the tests or reporting the results of those tests, if any, here. If not reported, write “not reported”. If unclear, write “unclear”. |  |  |
| 48d. For untestable assumptions (e.g. unmeasured confounding), is there anything the authors do to evaluate the plausibility of those assumptions (e.g. negative control exposures or outcomes, quantitative bias analysis)? If yes, which ones? Copy and paste relevant text describing the tests or reporting the results of those tests, if any, here. If not reported, write “not reported”. If unclear, write “unclear”. |  |  |
| 49. Do the authors report any use of weighting? |  |  |
| 50. Did the authors investigate the potential for heterogeneity in causal effects?    If YES, did the authors discuss heterogeneity of estimated causal effects and the possible impact on the generalizability of research findings? |  |  |
| 51. Sensitivity analyses |  |  |

| 52. Notes |  |  |
| --- | --- | --- |
