# Supplementary material for: Application of causal inference methods in individual-participant data meta-analyses in medicine: addressing data handling and reporting gaps with new proposed reporting guidelines
Source: BMC Med Res Methodol. 2024 Apr 19;24:91. doi: 10.1186/s12874-024-02210-9 (PMC11027270; doi:10.1186/s12874-024-02210-9)
Supplement: Supplementary file 4 — Supplementary Material 4. [file 12874_2024_2210_MOESM4_ESM.docx]

Supplementary Material 4. Included Studies

| Study | #/types studies | Exposures | Health outcome? | #countries | #participants pooled |
| --- | --- | --- | --- | --- | --- |
| Hansson | 8 cohorts | snus use | Incidence and survival after Stroke (overall and subtypes: Ischaemic, Haemorrhagic, unspecified) r | Sweden | 130,485 |
| Dilworth | 2 observational studies | Treatment with Vancomycin Treatment with Vancomycin combined with β-lactam class ((penicillin, cephalosporin, and carbapenem) (combo) | primary outcome was persistent bacteremia | USA | 177 |
| Kuramatsu | 4 cohorts | Surgical Hematoma Evacuation vs. Conservative Treatment | functional disability evaluated by the modified Rankin Scale ([mRS] score range: 0, no functional deficit to 6, death) at 3 months; favorable (mRS, 0-3) vs unfavorable (mRS, 4-6). | Germany, USA | 578 |
| Bastos | 31 cohorts | MDR and XDR TB treatments | MRT and XDR status | --  (28 countries across all WHO regions) | 9290 |
| Grams | 8 cohorts | apo L1 gene (APOL1) variants G1 and G2 | Cardiovascular disease | USA | 21,304 |
| Fretts | 12 cohorts | lipids | associations of circulating fatty acids of interest with nongenetic outcome | Europe and US | ~435,000 |
| Ding | 6 cohorts | angiotensin-converting enzyme inhibitors (ACEIs), angiotensin II receptor blocker (ARBs), β blockers, calcium channel blockers (CCBs; including long-acting and short- acting preparations), and diuretics" (antihypertensive medications) | dementia and Alzheimer's | usa, netherlands, iceland, france | ~31000 |
| Gibson | 5 cohorts | BMI (main)  race/ethnicity, CRC stage, smoking status, physical activity, hormone replacement therapy use (confounders) | Second obesity-associated cancer (postmenopausal, breast, kidney, pancreas, esophageal adenocarcinoma, endometrium) | USA | 567,169 (pulled from citations) |
| Martin | 5 RCTs and 1 observational cohort | Condom use | HSV-2 Acquisition | USA, Australia, North America, Europe, Latin America | 5384 |
| Jokela | 10 prospective cohorts | Lower conscientiousness | Mortality, disability | Britain, USA, Australia, Germany | ~158,873 |
| Hach | 7 cohorts | Non-randomized:  Education  Smoking  Alcohol consumption | Tooth loss | Denmark | 84,843 |
| Leon | 3 prospective cohorts | Non-randomized: 14 pesticide chemical groups and 33 active ingredients of each group  OP Insecticides - Chlorpyrifos - Dichlorvos - Malathion - Parathion - Terbufos Carbamate insecticides - Aldicarb - Carbaryl - Carbofuran - Pirimicarb OC insecticides - DDT - Lindane Pyrethroid insecticides - Deltamethrin - Esfenvalerate - Permethrin (Phenyl) urea herbicides - Isoproturon - Linuron Dicamba Chloroacetanilides - Alachlor - Metolachlor Dinitroaniline herbicides - Trifluralin  Glyphosate  Phenoxy herbicides  - 2, 4-D  - MCPA  - MCPP  Thiocarbamate herbicides  - Butylate  - EPTC  Triazine herbicides  - Atrazine  - Simazine  Triazinone herbicides  - Metribuzin  Dithiocarbamate fungicides  - Mancozeb  - Thiram  Phthalimide fungicides  - Captafol  - Captan  Arsenicals | Non-Hodgkin lymphoid malignancies  Chronic lymphocytic leukaemia/small lymphocytic lymphoma  Diffuse large B-cell lymphoma  Follicular lymphoma  Multiple myeloma/plasma-cell leukaemia | France, Norway, USA | 316,270 |
| Rota | 2 nested cohorts, 12 selected controls from the general population, 11 hospital-based case-control studies | Non-randomized:  Socioeconomic position | Gastric cancer (C16.0–C16.9);  Further subclassified in gastric cardia cancer (ICD-10 C16.0) and noncardia cancers (ICD-10 C16.1–C16.9) | Greece, Italy, Portugal, Russia, Spain, Sweden, China, Iran, Canada, USA, Brazil, Mexico | 34,146 |
| Mondul | 5 prospective cohorts, 4 randomized controlled trials | Non-randomized:  Four SNPs  rs2282679 (GC)  rs6013897 (CYP2R1)  rs10741657 (DHCR7)  rs12785878 (CYP24A1) | Breast cancer | Denmark, Great Britain, Germany, Greece, Italy, the Netherlands, Spain, Sweden | ~553,676 |
| Elke | 2 prospective cohorts | Non-randomized:  Energy intake through enteral nutrition  Protein intake through enteral nutrition | 60-day mortality  Ventilator-free days | --  (33 countries) | 2270 |
| Peres | 6 prospective cohorts | Non-randomized:  C-Reactive Protein | Pithelial ovarian cancer | USA, Europe | 3042 |
| Crowe | Number: 7  Type:  2 case-control studies nested within a prospective study  3 case-control studies nested within a RCT  1 case-cohort study nested within a prospective study  1 case-cohort design nested within a RCT | Non-randomized:  Fraction measured  Saturated fatty acids  Myristic acid  Pentadecanoic acid  Heptadecanoic acid  Stearic acid   Monounsaturated fatty acids  Palmitoleic acid  Oleic acid   Total n-3 polyunsaturated acids  α-linolenic acid  Eicosapentaenoic acid  n-3 docosapentaenoic acid  Docosahexaenoic acid   Total n-6 polyunsaturated acids  Linoleic acid  Dihomo-γ-linolenic acid  Arachidonic acid | prostate cancer | USA, Europe, Australia, North America | 11,747 |
| Bosetti | 15 case-control studies | Non-randomized:  History of diabetes  Antidiabetic medications | Pancreatic cancer | USA, Europe, Greece, Italy, Australia, Canada | 22,292 |
| Keller | 5 prospective cohorts, 2 RCTs | Non-randomized exposure:  Sugar-sweetened beverages (“carbonated/non‐carbonated and caffeinated/non-caffeinated sodas, sport drinks and fruit drinks with any type of added sugar”)  Caffeinated coffee (“all types of plain (unsweetened) coffee with caffeine and total coffee also included decaffeinated coffee”)  Tea (“all types of plain (unsweetened) tea”)  Milk (“non-sweetened cow milk, either whole-fat, low-fat or total milk (whole-fat and low-fat combined“)  Fruit juice (“100% fruit juice“)  artificially-sweetened beverages (“any diet drinks sweetened with artificial sweeteners”) | fatal coronary heart disease  nonfatal myocardial infarction | USA, Finland | 284,345 |
| Cook | 11 case-control studies, 8 cohorts, 2 RCTs | Non-randomized:  Cigarette smoking (status, intensity, duration, pack-years, age at initiation; ever smoked pipe or cigars, chewed tobacco, snuff)  Alcohol consumption (gram per day, type of drink) | Male breast cancer | Europe (Denmark, France, Germany, Italy, Latvia, Portugal, Spain, Sweden, Greece, Norway, United Kingdom), Canada, USA | 5,266,749 |
| Bethea | 7 prospective cohorts | Non-randomized:  Body Mass Index | Pancreatic cancer mortality | USA | 239,596 |
| Rogozinska | 36 RCTs | Non-randomized:  Gestational weight gain | Adverse maternal and offspring outcomes:  Any type of caesarean section  Large for Gestational Age (LGA) infant  Small for Gestational Age (SGA) infant  Preterm delivery (before 37 week of gestation) | Netherlands, USA, Spain, Belgium, Brazil, Australia, Egypt, Canada, Norway, Iran, Finland, Germany, Denmark, Ireland | 9373 |
| Yang | 20 prospective cohorts | Tobacco Use Status, age at starting smoking, number of cigarettes smoked per day, and age at quitting smoking | Country of origin and birth cohort-specific mortality and the population attributable risk for deaths from all causes and from lung cancer. | China, Japan, Korea, Singapore, Taiwan, India | 1,002,358 |
| Yamamoto | 1 cohort, 2 RCTs | Non-randomised:  Chronic Kidney Disease  Atrial Fibrillation  Antiplatelet and antithrombotic therapies   Randomised (only in the RCTs):  PCI with different types of drug-eluted stents | Bleeding (intracranial, abdominal or other location) or thrombotic (MI or stroke) events | Japan | 22,380 |
| Voerman | 37 cohorts | Pre-pregnancy  Gestational weight gain | Childhood overweight/obesity | Netherlands, UK, Canada, Sweden, Italy, Denmark, France, Ukraine, Greece, Germany, Norway, Spain, Poland, Finland, USA, Australia, Ireland, Slovak Republic | 162,129 |
| Gall | 2 cohorts | Parental smoking | Carotid intima-media thickness | Finland, Australia | 1930 (comment mentions different numbers) |
| Marklund | 31 studies   PC prospective cohort  RCC retrospective case-control  PNC prospective nested case-cohort  PCC prospective nested case-control | Omega-6 Fatty Acids | Cardiovascular disease incidence and mortality | Iceland, USA, Taiwan, Costa Rica, Denmark, UK, Germany, Finland, Australia, Netherlands, Sweden, France, Singapore, Japan | ~559,039 (numbers only mentioned in supplemental table) |
| Sun | 1 randomized control trial, 2 cohorts | Treatment received: CHM or CT | Survival time | China | 486 |
| Danforth | 2 Prospective cohorts | Type 2 Diabetes | Urinary Incontinence | USA | 71,650 |
