# Supplementary material for: Application of causal inference methods in individual-participant data meta-analyses in medicine: addressing data handling and reporting gaps with new proposed reporting guidelines
Source: BMC Med Res Methodol. 2024 Apr 19;24:91. doi: 10.1186/s12874-024-02210-9 (PMC11027270; doi:10.1186/s12874-024-02210-9)
Supplement: Supplementary file 5 — Supplementary Material 5. [file 12874_2024_2210_MOESM5_ESM.docx]

Supplementary Material 5. Proposed Reporting Guidelines

| Item No. | Section | Checklist Item |
| --- | --- | --- |
| Title and Abstract | | |
| 1 | Title | State that the study is an IPD-MA, and which type causal method was implemented (e.g., Instrumental Variable, Mediation Analysis, Regression Discontinuity) |
| 2 | Abstract |  |
|  | a | Identify which type of meta-analysis technique (one-step or two-step) was implemented |
|  | b | Objectives, including the research questions, and listing participants, interventions, comparators, and outcomes |
|  | c | Methods: clearly state the inclusion/exclusion criteria; sources of information and how they were found (e.g., search strategy and data bases, or contact with study PI); how one assessed risk of bias, which pooling technique was selected; which type causal method implemented (e.g., Instrumental Variable, Mediation Analysis, Regression Discontinuity) |
|  | d | Results: number of included studies; description and number of participants in final analysis; direction, size and magnitude of the effect(s) |
|  | e | Discussion: strengths and limitations of evidence; interpretation of results |
| Introduction | | |
| 3 | Background | - Describe the study background and rationale for investigating the mechanism(s) of interest - Include supporting evidence or rationale for why the intervention or exposure might have a causal relationship with the outcome(s) - Include supporting evidence or rationale for why the selected causal method is appropriate for the data and study question |
| 4 | Objectives | - Cleary state the research question(s) being addressed, with references, if applicable, to why this would be relevant to the participants, interventions, outcomes, etc. chosen - Clearly state hypotheses (if any) - State that the causal method selected is intended to estimate causal effects if specific assumptions are met |
| Methods | | |
| 5 | Study Design and Data Sources | Present key elements of the study design phases |
|  | a | Setting: Describe how the studies were identified; the inclusion/exclusion criteria; the study designs of the studies included;  Describe the location and dates of data collection of each population included in the analysis, including the recruitment period, the follow-up period, how many time-points (baseline + follow-up) are included in the analysis. |
|  | b | Participants: Describe the characteristics of the participants included in the final analysis, inclusion and exclusion criteria; and any sample size calculations that were carried out a priori. |
|  | c | IPD integrity: describe which aspects of the IPD were subject to data checking (such as sequence generation, data consistency and completeness, baseline imbalance) and how this was done |
|  | d | Describe the measurement, quality control, and selection of exposures, confounders, and outcome variables, as well as quasi-experimental variables (thresholds for Regression Discontinuity, and Instruments for Instrumental Variable Analyses). Describe if variables differed in their measurement or definitions in the original studies, and how this dealt with. Describe adjustments made in harmonization to pool these variables (if applicable). |
|  | e | For all of the above variables, clearly state which of them were randomized or non-randomized in each of the original studies, and how you accounted for differences when pooling them. |
|  | f | Risk of Bias: Describe methods used to assess risk of bias in the individual studies and whether this was applied separately for each outcome. If applicable, describe how findings of IPD checking were used to inform the assessment. |
|  | g | Provide details of ethics committee approval and/or informed consent from participants, if relevant |
| 6 | Assumptions | Explicitly state the assumptions required for the causal method implemented. |
|  | a | State name(s) of testable assumption(s), and how they were tested. |
|  | b | Include a graphic representation of the assumed causal model. |
| 7 | Statistical Methods, Main Analysis |  |
|  | a | Describe the estimation method used for deriving the (pooled) causal effect. |
|  | b | Specify for which parameter the estimation method is being used. Multiple estimation methods can be used for different parameters (e.g. estimation of propensity model versus estimation of analysis model). |
|  | b | State which type of effect is estimated (marginal, conditional). |
| 8 | Missing Data | Describe types of missingness, and how it was accounted for. |
|  | a | Describe presence of missing data within and across studies (e.g. presence of sporadically and systematically missing values). |
|  | b | Did the study describe possible reasons/mechanisms of missingness |
|  | c | Describe how missing data within and across studies was accounted for. Specifically, describe the method that was used for the primary analysis (e.g. omission of patients with missing values or multiple imputations). |
|  | d | In case imputation was used, describe what variables were included in the imputation model and why. |
|  | e | In case of imputation, describe efforts that were made to account for potential heterogeneity between studies (e.g. impute each study separately, or adopt multilevel imputation methods). |
| 9 | Pooling | Describe the meta-analysis methods used to synthesize IPD. Specify any statistical methods and models used. Issues should include (but are not restricted to): |
|  | a | Describe which approach was used to pool data (one-stage or two-stage). |
|  | b | Describe how effect estimates were generated separately within each study and combined across studies (where applicable). |
|  | c | Specify one-stage models (where applicable), including how clustering of patients within studies was accounted for. |
|  | d | Describe use of fixed or random effects models and any other model assumptions, such as proportional hazards. |
|  | e | Describe how (summary) survival curves were generated (where applicable). |
|  | f | Describe methods for quantifying statistical heterogeneity (such as I^2^ and τ^2^ ). |
|  | g | Describe how studies providing IPD and not providing IPD were analyzed together (where applicable). |
|  | h | Describe how missing data within the IPD were dealt with (where applicable). |
| 10 | Sensitivity Analyses | Describe any sensitivity analyses or additional analyses performed (e.g., comparison of effect estimates from different approaches, independent replication, bias analytic techniques, validation of instrument, simulation). |
| 11 | Software and Preregistration |  |
|  | a | Name all statistical software and package(s), including version and settings used. |
|  | b | Protocol & Registration: indicate if a study protocol exists and where it can be accessed; indicate if there were any deviations from the protocol; include search strategies implemented (such that it could be replicated), and clearly state how one obtained unpublished data if relevant. |
| Results |  |  |
| 12 | Main Results |  |
|  | a | Report point estimates and uncertainty estimates for the prespecified causal relationships. |
|  | b | Include a flow char with descriptive information (i.e., participants, events/cases, controls, exposed/treated, unexposed/non-treated), and time-points of each included in the analysis. |
|  | c | If inference concerning the causal relationship of interest is considered feasible given the causal assumptions, report the point estimate and uncertainty estimate. |
|  | d | If relevant, consider translating estimates of relative risk into absolute risk for a meaningful time period. |
|  | e | Consider plots to visualize results (e.g. forest plot, scatterplot of associations between genetic variants and outcome versus between genetic variants and exposure). |
| 13 | Assessment of Assumptions |  |
|  | a | State name(s) of the untestable assumption(s), and how they were evaluated (e.g., what attempts were made to invalidate the assumption). |
|  | b | Report any additional statistics required (e.g., F-statistics, I^2^, Q statistic, or E-value). |
| 13 | Sensitivity Analyses |  |
|  | a | Report any results of the sensitivity analyses undertaken (e.g., assess the robustness of the main results to violations of the assumptions). |
|  | b | Report results from any other sensitivity analyses or additional analyses |
|  | c | If relevant, report any assessment of the direction of the causal relationship (e.g., bidirectional Mendelian Randomization). |
|  | d | If relevant, report and compare with estimates from analyses which did not implement causal methodologies. |
|  | e | Consider plots to visualize results (e.g., forest plot, scatterplot of associations between genetic variants and outcome vs between genetic variants and exposure when using Mendelian Randomization; or plot of data points around a threshold when using Regression Discontinuity). |
| Discussion | | |
| 12 | Key Results | Summarize key results with reference to study objectives. |
| 13 | Limitations | Discuss limitations of the study including potential sources of bias, imprecision. Take assumptions into account. Discuss direction and magnitude of potential bias and any efforts to address them. |
| 14 | Interpretation |  |
|  | a | Meaning: Provide a general interpretation of the findings in the context of other evidence. |
|  | b | Mechanism: Discuss underlying biological mechanisms that could drive a potential causal relationship between the investigated exposure and the outcome, and whether the gene-environment equivalence assumption is reasonable. Use causal language carefully, clarifying that the causal estimates may provide causal effects only under certain assumptions. |
|  | c | Clinical relevance: Discuss whether the results have clinical or public policy relevance, and to what extent they inform effect sizes of possible interventions. |
| 15 | Generalizability |  |
|  | a | Clearly state any investigation into the potential for heterogeneity in causal effects. |
|  | b | Discuss the generalizability of the study results (a) to other populations, (b) across other exposure periods/timings, and (c) across other levels of exposure (if applicable). |
| Other Information | | |
| 16 | Funding | Describe sources of funding and the role of funders in the present study and, if applicable, sources of funding for the databases and original study or studies on which the present study is based. |
| 17 | Data and Data sharing | Provide the data used to perform all analyses or report where and how the data can be accessed, and reference these sources in the article. Provide the statistical code needed to reproduce the results in the article, or report whether the code is publicly accessible and if so, where applicable. |
| 18 | Conflicts of interest | State any conflicts of interest and financial disclosures for all authors. |
