# Supplementary material for: Application of causal inference methods in individual-participant data meta-analyses in medicine: addressing data handling and reporting gaps with new proposed reporting guidelines
Source: BMC Med Res Methodol. 2024 Apr 19;24:91. doi: 10.1186/s12874-024-02210-9 (PMC11027270; doi:10.1186/s12874-024-02210-9)
Supplement: Supplementary file 6 — Supplementary Material 6. [file 12874_2024_2210_MOESM6_ESM.docx]

Supplementary Material 6. Reporting Guidelines Comparison

| Section/Topic | Item | PRISMA-IPD Item Checklist  PRISMA-IPD Statement | | Mediation Analysis  AGReMA Statement | Mendelian Randomization  Strobe-MR Statement | Pooled Studies Using Quasi-Experimental Methods (for pooled studies with causal intent)  (This Current Review) |
| --- | --- | --- | --- | --- | --- | --- |
| Structured  Abstract/  Summary | 1 | TITLE | 1. Title: Identify the report as a systematic review, meta- analysis, or both | 1. Identify that the study uses mediation analyses | 1. Title— Indicate mendelian randomisation (MR) as the study’s design in the title and/or the abstract if that is a main purpose of the study. | Title |
|  | 2 | BACK GROUND | 2. Objectives: The research question including components such as participants, interventions, comparators, and outcomes. | 2. Abstract—  - Provide a structured summary of the objectives, methods, results, and conclusions specific to mediation analyses |  | Objectives, health outcomes. |
|  |  | METHODS | 3. Eligibility criteria: Study and report characteristics used as criteria for inclusion. 4. Information sources: Key databases searched and search dates. 5. Risk of bias: Methods of assessing risk of bias. |  |  | Types of studies included. Randomized and non-randomized studies. |
|  |  | RESULTS | 6. Included studies: Number and type of included studies and participants and relevant characteristics of studies. 7. Synthesis of results: Results for main outcomes (benefits and harms), preferably indicating the number of studies and participants for each. If meta-analysis was done, include summary measures and confidence intervals. 8. Description of the effect: Direction of the effect (i.e., which group is favored) and size of the effect in terms meaningful to clinicians and patients. Describe of the direction and size of summary effects in terms meaningful to those who would put findings into practice. report eligibility criteria; data sources including dates of last bibliographic search or elicitation, noting that IPD were sought; methods of assessing risk of bias |  |  | Does the study attempt to establish a causal relationship? If 1) causal inference methods (as described in the protocol; e.g., regression  discontinuity design, instrumental variable approach,  G-methods) were used,  or 2) the language suggests a causal intent AND the regression-based analysis (e.g., Cox proportional hazards model) adjusted for a set of possible confounders, include. If the aim of the study is descriptive, predictive, or prognostic, exclude. If uncertain, label as uncertain.  Effect size estimated. |
|  |  | DISCUSSION | 9. Strengths and Limitations of evidence: Brief summary of strengths and limitations of evidence (e.g., inconsistency, imprecision, indirectness, or risk of bias, other supporting or conflicting evidence). 10. Interpretation: General interpretation of the results and important implications. provide number and type of studies and participants identified and number (%) obtained; summary effect estimates for main outcomes (benefits and harms) with confidence intervals and measures of statistical heterogeneity. state main strengths and limitations of the evidence, general interpretation of the results and any important implications |  |  |  |
| Rationale  (Main Manuscript) | 3 | Describe the rationale for the review in the context of what is already known. | | 3. Background and Rationale—  - Describe the study background and theoretical rationale for investigating the mechanisms of interest  - Include supporting evidence or theoretical rationale for why the intervention or exposure might have a causal relationship with the proposed mediators  - Include supporting evidence or theoretical rationale for why the mediators might have a causal relationship with the outcomes | 2. Background—  Explain the scientific background and rationale for the reported study. What is the exposure? Is a potential causal relationship between exposure and outcome plausible? Justify why MR is a helpful method to address the study question. |  |
| Objectives | 4 | Provide an explicit statement of the questions being addressed with reference, as applicable, to participants, interventions, comparisons, outcomes and study design (PICOS). Include any hypotheses that relate to particular types of participant-level subgroup | | 4. Objectives—  - State the objectives of the study specific to the mechanisms of interest  - The objectives should specify whether the study aims to test or estimate the mechanistic effects | 3. Objectives— State specific objectives clearly, including pre-specified causal hypotheses (if any). State that MR is a method that, under specific assumptions, intends to estimate causal effects | 1. Health outcomes 2. Includes at least one non-randomized exposure variable 3. Clearly stated causal intent |
| Protocol & Registration | 5 | Indicate if a protocol exists and where it can be accessed. If available, provide registration information including registration number and registry name. Provide publication details, if applicable. | | 5. Study registration—  If applicable, provide references to any protocols or study registrations specific to mediation analyses and highlight any deviations from the planned protocol | 4. Study design and data sources—  4a) Setting Describe the study design and the underlying population, if possible. Describe the setting, locations, and  relevant dates, including periods of recruitment, exposure, follow-up, and data collection, when available  4b) Participants  Report the eligibility criteria and the sources and methods of selection of participants. Report the sample size and whether any power or sample size calculations were carried out prior to the main analysis.  4c)  Describe measurement, quality control, and selection of genetic variants.  4d)  For each exposure, outcome, and other relevant variables, describe methods of assessment and diagnostic criteria for diseases.  4e)  Provide details of ethics committee approval and participant informed consent, if relevant. |  |
|  |  |  |  |  |  |  |
| Eligibility Criteria | 6 | Specify inclusion and exclusion criteria including those relating to participants, interventions, comparisons, outcomes, study design and characteristics (e.g. years when conducted, required minimum follow-up). Note whether these were applied at the study or individual level i.e. whether eligible participants were included (and ineligible participants excluded) from a study that included a wider population than specified by the review inclusion criteria. The rationale for criteria should be stated. | | 7. Participants—  - Describe the target population, eligibility criteria specific to mediation analyses, study locations, and study dates (start of participant enrollment and end of follow-up) |  | 1. Focused on health outcomes 2. Parent study composed of 2 separate studies |
|  |  |  |  |  |  |  |
|  |  |  |  |  |  |  |
| Information sources  Identifying studies | 7 | Describe all methods of identifying published and unpublished studies including, as applicable: which bibliographic databases were searched with dates of coverage; details of any hand searching including of conference proceedings; use of study registers and agency or company databases; contact with the original research team and experts in the field; open advertisements and surveys. Give the date of last search or elicitation | | 6. Study design and source of data—  - Specify the design of the original study that was used in mediation analyses and where the details can be accessed, supported by a reference  - If applicable, describe study design features that are relevant to mediation analyses |  | - List number & types of individual studies or cohorts included in the pooled analysis (e.g. 3 cohort studies, 2 case-control studies, patient registries) - Study populations of each individual study or cohort included in the pooled analysis - Number of participants in each individual study or cohort included in the pooled analysis - Recruitment period of each individual study or cohort included in the pooled analysis - Location of data collection of each individual study or cohort included in the pooled analysis |
| Search  Identifying studies—search | 8 | Present full electronic search strategy for at least one database, including any limits used, such that it could be repeated | |  |  |  |
| Study selection | 9 | State the process for determining which studies were eligible for inclusion | |  |  |  |
| Data collection process | 10 | Describe how IPD were requested, collected and managed, including any processes for querying and confirming data with investigators. If IPD were not sought from any eligible study, the reason for this should be stated (for each such study). If applicable, describe how any studies for which IPD were not available were dealt with. This should include whether, how and what aggregate data were sought or extracted from study reports and publications (such as extracting data independently in duplicate) and any processes for obtaining and confirming these data with investigators | |  |  |  |
| Data items | 11 | Describe how the information and variables to be collected were chosen. List and define all study level and participant level data that were sought, including baseline and follow-up information. If applicable, describe methods of standardizing or translating variables within the IPD datasets to ensure common scales or measurements across studies | |  |  | - Location of data collection of each individual study or cohort included in the pooled analysis  - What exposures are studied in “parent” study? List which are randomized and which are non-randomized.  - What are the primary outcomes in the “parent” study? (e.g. myocardial infarction, hypertension, remission) |
| IPD Integrity | 12 | Describe what aspects of IPD were subject to data checking (such as sequence generation, data consistency and completeness, baseline imbalance) and how this was done | |  |  | - Did the study describe how variables were measured and defined in each study?  - Did the study describe any differences in measurement and definition of variables across studies?  - If the answer to the above is yes, did the study discuss how it dealt with differences in variable definitions and measurement methods (standardization or harmonization)? |
| Risk of bias in individual studies | 13 | Describe methods used to assess risk of bias in the individual studies and whether this was applied separately for each outcome. If applicable, describe how findings of IPD checking were used to inform the assessment. Report if and how risk of bias assessment was used in any data synthesis. | |  |  |  |
| Summary measures  Specification of outcomes and effect measures | 14 | State all treatment comparisons of interests. State all outcomes addressed and define them in detail. State whether they were pre-specified for the review and, if applicable, whether they were primary/main or secondary/additional outcomes. Give the principal measures of effect (such as risk ratio, hazard ratio, difference in means) used for each outcome. | |  |  |  |
| Synthesis of results  Synthesis methods | 15 | Describe the meta-analysis methods used to synthesize IPD. Specify any statistical methods and models used. Issues should include (but are not restricted to): - Use of a one-stage or two-stage approach. - How effect estimates were generated separately within each study and combined across studies (where applicable). - Specification of one-stage models (where applicable), including how clustering of patients within studies was accounted for. - Use of fixed or random effects models and any other model assumptions, such as proportional hazards. - How (summary) survival curves were generated (where applicable). - Methods for quantifying statistical heterogeneity (such as I 2 and τ2 ). - How studies providing IPD and not providing IPD were analyzed together (where applicable). - How missing data within the IPD were dealt with (where applicable). | |  |  | - Do the authors discuss any of the assumptions required for the analysis methods they have chosen to pool the data? If yes, which ones? Copy and paste relevant text describing the tests or reporting the results of those tests, if any, here. If not reported, write “not reported”. If unclear, write “unclear”.  - What estimation method was used for deriving the (pooled) causal effect? Specific for which parameter the estimation method is being used Multiple estimation methods can be used for different parameters (e.g. estimation of propensity model versus estimation of analysis model).  - Are authors estimating a marginal or conditional effect? (yes, no, unclear)  - Pooled analysis – What type of estimand is used?  - Do the authors report testing any of the assumptions required for the analysis methods they have chosen to pool the data? If yes, which ones?  - Did the authors analyze each dataset separately and pooled the corresponding results? (or did they analyze all data directly using a so-called one-stage approach?)  - If YES, please define what method was used to pool results across studies (e.g. random effects meta-analysis)  - If NO, go to question 44a  - List approach(es) to account for clustering/ heterogeneity at the cohort or pooled study level (whichever units are pooled across) (note whether this is done to stratify within or across studies)  - How did they adjust for (potential) heterogeneity in baseline risk, confounder effects, mediator effects, causal effects etc.?  - Did the study describe the presence of missing data within and across studies? (e.g. presence of sporadically and systematically missing values)  - Did the study describe possible reasons/mechanisms of missingness?  - How did the study account for missing data within and across studies? Specifically, what method was used for the primary analysis? (e.g. omission of patients with missing values or multiple imputations)  - In case imputation was used, do the authors discuss what variables were included in the imputation model and why?  - In case of imputation, what efforts were made to account for potential heterogeneity between studies? (e.g. impute each study separately, or adopt multilevel imputation methods) |
| Causal Methods |  |  | | 9. Effects of interest—  - specify the effects of interest  10. Assumed causal model—  - Include graphic representation of assumed causal model including the exposure, mediator, outcome, and possible confounders  11. Causal assumptions—  - specify assumptions about the causal model  12. Measurement—  - Clearly describe the interventions or exposures, mediators, outcomes, confounders, and moderators that were used in the analyses  - Specify how and when they were measured, the measurement properties, and whether blinded assessment was used  13. Measurement levels—  - If relevant, describe the levels at which the exposure, mediator, and outcome were measured  14. Statistical Methods—  - Describe the statistical methods used to estimate the causal relationships of interest  - This description should specifcy analytic strategies used to reduce confounding, model building procedures, justification for the inclusion or exclusion of possible interaction terms, modeling assumptions, and methods used to handle missing data  - Provide a reference to the statistical software and package used | 5. Assumptions— Explicitly state the 3 core instrumental variable (IV) assumptions for the main analysis (relevance, independence and exclusion restriction), as well assumptions for any additional or sensitivity analysis | - Which covariates were adjusted for in the analysis? (e.g. by considering them as adjustment in regression, or as a variable of propensity score model, or as a matching variable)  - Were they labeled as confounders or mediators of the causal relationships? If yes, list them.  - On what basis were the confounders selected? Studies may have restricted to a set of confounders because those were the most commonly measured variables across studies, or defined a list of confounders based on a directional acyclic graph (DAG) and imputed study level information for systematically missing confounders; or combined fully and partially adjusted studies in a multivariate approach.  - Which methods were used with the pooled data to make causal inferences? (e.g. interrupted time series with a control group; comparative study without concurrent controls; IV; Mendelian randomization; RD; interrupted time series, including DiD estimation; G-estimation; multiple regression adjusting for confounders; propensity score matching; inverse probability of treatment weighting)  - Justification for method(s) used (e.g. “we selected a synthetic control approach because this method is well-suited to situations involving 1 intervention unit, and many controls and may better approximate counterfactual post-intervention outcomes than using any single control or an evenly weighted combination of controls” or “This approach is advantageous, because characteristics of each region, other than the occurrence of the treatment, are unlikely to change appreciably over so short a time period. Thus, each region serves as its own control, allowing us to control for other community-level characteristics that may also be associated with injuries.”)  - Did the authors explicitly state the assumptions required for causal inference methods? If yes, which ones? (e.g. ignorability, positivity, stable unit treatment value, transitivity) Copy and paste relevant text, if any, here. If not reported, write “not reported”. If unclear, write “unclear”.  - Do the authors report testing any of the testable assumptions required for the analysis methods they have chosen to deliver causal effects? If yes, which ones? Copy and paste relevant text describing the tests or reporting the results of those tests, if any, here. If not reported, write “not reported”. If unclear, write “unclear”.  - For untestable assumptions (e.g. unmeasured confounding), is there anything the authors do to evaluate the plausibility of those assumptions (e.g. negative control exposures or outcomes, quantitative bias analysis)? If yes, which ones? Copy and paste relevant text describing the tests or reporting the results of those tests, if any, here. If not reported, write “not reported”. If unclear, write “unclear”.  - Do the authors report any use of weighting? |
|  |  |  |  |  | 6. Statistical methods: main analysis—  Describe statistical methods and statistics used  6a) Describe how quantitative variables were handled in the analyses (ie, scale, units, model)  6b) Describe how genetic variants were handled in the analyses and, if applicable, how their weights were selected  6c) Describe the MR estimator (eg, 2-stage least squares, Wald ratio) and related statistics. Detail the included covariates and, in case of 2-sample MR, whether the same covariate set was used for adjustment in the 2 samples.  6d) Explain how missing data were addressed  6e) If applicable, indicate how multiple testing was addressed |  |
| Exploration of variation in effects | 16 | If applicable, describe any methods used to explore variation in effects by study or participant level characteristics (such as estimation of interactions between effect and covariates) State all participant-level characteristics that were analyzed as potential effect modifiers, and whether these were pre-specified. | |  |  | Which covariates were adjusted for in the analysis? (e.g. by considering them as adjustment in regression, or as a variable of propensity score model, or as a matching variable)  - Were they labeled as confounders or mediators of the causal relationships? If yes, list them.  - On what basis were the confounders selected? Studies may have restricted to a set of confounders because those were the most commonly measured variables across studies, or defined a list of confounders based on a directional acyclic graph (DAG) and imputed study level information for systematically missing confounders; or combined fully and partially adjusted studies in a multivariate approach |
| Risk of bias across studies | 17 | Specify any assessment of risk of bias relating to the accumulated body of evidence, including any pertaining to not obtaining IPD for particular studies, outcomes or other variables | |  |  |  |
| Additional Analyses | 18 | Describe methods of any additional analyses, including sensitivity analyses. State which of these were pre-specified | | 15. Sensitivity Analyses—  - Describe any sensitivity analyses that were used to explore causal or statistical assumptions and the influence of missing data | 8. Describe any sensitivity analyses or additional analyses—  Describe any sensitivity analyses or additional analyses performed (e.g. comparison of effect estimates from different approaches, independent replication, bias analytic techniques, validation of instruments, simulations) | Were Sensitivity analyses conducted? |
|  |  |  |  | 16. Ethical approval—  - Name the institutional research board or ethics committee that approved the study  - Provide a description of participant informed consent or ethics committee waiver of informed consent. | 9) Software and pre-registration—  a) Name statistical software and package(s), including version and settings used b) State whether the study protocol and details were pre-registered (as well as when and where) |  |
| Study Selection | 19 | Give numbers of studies screened, assessed for eligibility, and included in the systematic review with reasons for exclusions at each stage. Indicate the number of studies and participants for which IPD were sought and for which IPD were obtained. For those studies where IPD were not available, give the numbers of studies and participants for which aggregate data were available. Report reasons for non-availability of IPD. Include a flow diagram | | 17. Participants—  - Describe baseline characteristics of participants included in mediation analyses  - Report the total sample size and number of participants los during follow-up or with missing data | 10. Descriptive data results—  a) Report the numbers of individuals at each stage of included studies and reasons for exclusion. Consider use of a flow diagram b) Report summary statistics for phenotypic exposure(s), outcome(s), and other relevant variables (e.g. means, SDs, proportions) c) If the data sources include meta-analyses of previous studies, provide the assessments of heterogeneity across these studies d) For 2-sample MR:  i. Provide justification of the similarity of the genetic variant-exposure associations between the exposure and outcome samples  ii. Provide information on the number of individuals who overlap between the exposure and outcome studies | Number and types of studies included in the pooled analysis |
| Study characteristics | 20 | For each study, present information on key study and participant characteristics (such as description of interventions, numbers of participants, demographic data, unavailability of outcomes, funding source, and if applicable duration of follow-up). Provide (main) citations for each study. Where applicable, also report similar study characteristics for any studies not providing IPD. | |  |  | - Study populations of each individual study included in the pooled analysis  - Number of participants in each individual study  - Recruitment period of each study  - Location of data collection of each individual study |
| IPD Integrity | 21 | Report any important issues identified in checking IPD or state that there were none. | |  |  |  |
| Risk of bias within studies | 22 | Present data on risk of bias assessments. If applicable, describe whether data checking led to the up-weighting or down-weighting of these assessments. Consider how any potential bias affects the robustness of meta-analysis conclusions. | |  |  |  |
| Results of individual studies | 23 | For each comparison and for each main outcome (benefit or harm), for each individual study report the number of eligible participants for which data were obtained and show simple summary data for each intervention group (including, where applicable, the number of events), effect estimates and confidence intervals. These may be tabulated or included on a forest plot. | | 18. Outcomes and estimates—  - Report point estimates and uncertainty estimates for the exposure-mediator and mediator-outcome relationships  - If inference concerning the causal relationship of interest is considered feasible given the causal assumptions, report the point estimate and uncertainty estimate |  |  |
| Synthesis of results  Results of syntheses | 24 | Present summary effects for each meta-analysis undertaken, including confidence intervals and measures of statistical heterogeneity. State whether the analysis was pre-specified, report the numbers of studies and participants, and, where applicable, the number of events on which it is based. When exploring variation in effects due to patient or study characteristics, present summary interaction estimates for each characteristic examined, including confidence intervals and measures of statistical heterogeneity. State whether the analysis was pre-specified. State whether any interaction is consistent across trials. Provide a description of the direction and size of effect in terms meaningful to those who would put findings into practice. | |  | 11. Main results—  a) Report the associations between genetic variant and exposure, and between genetic variant and outcome, preferably on an interpretable scale b) Report MR estimates of the relationship between exposure and outcome, and the measures of uncertainty from the MR analysis, on an interpretable scale, such as odds ratio or relative risk per SD difference c) If relevant, consider translating estimates of relative risk into absolute risk for a meaningful time period d) Consider plots to visualize results (e.g. forest plot, scatterplot of associations between genetic variants and outcome versus between genetic variants and exposure) |  |
|  |  |  |  |  | 12. Assessment of assumptions—  a) Report the assessment of the validity of the assumptions b) Report any additional statistics (e.g., assessments of heterogeneity across genetic variants, such as I2, Q statistic or E-value) |  |
| Risk of bias across studies | 25 | Present results of any assessment of risk of bias relating to the accumulated body of evidence, including any pertaining to the availability and representativeness of available studies, outcomes or other variables. | |  |  |  |
| Additional Analysis | 26 | Give results of any additional analyses (e.g. sensitivity analyses). If applicable, this should also include any analyses that incorporate aggregate data for studies that do not have IPD. If applicable, summarize the main meta-analysis results following the inclusion or exclusion of studies for which IPD were not available | | 19. Sensitivity Parameters—  - Report the results from any sensitivity analyses used to assess robustness of the causal or statistical assumptions and the influence of missing data | 13. Sensitivity analyses and additional analyses—  a) Report any sensitivity analyses to assess the robustness of the main results to violations of the assumptions b) Report results from other sensitivity analyses or additional analyses c) Report any assessment of direction of causal relationship (e.g., bidirectional MR) d) When relevant, report and compare with estimates from non-MR analyses e) Consider additional plots to visualize results (e.g., leave-one-out analyses) | Sensitivity analyses conducted |
| Summary of evidence | 27 | Summarize the main findings, including the strength of evidence for each main outcome | |  | Discussion—  14. Key results  Summarize key results with reference to study objective |  |
| Strengths and Limitations | 28 | Discuss any important strengths and limitations of the evidence. | | 20. Limitations—  - Discuss the limitations of the study including potential sources of bias | 15. Limitations—  Discuss limitations of the study, taking into account the validity of the IV assumptions, other sources of potential bias, and imprecision. Discuss both direction and magnitude of any potential bias and any efforts to address them. |  |
| Conclusions | 29 | Provide a general interpretation of the findings in the context of other evidence. | | 21. Interpretation—  - Interpret the estimated effects considering the study’s magnitude and uncertainty, plausibility of the causal assumptions, limitations, generalizability of the findings, and results from relevant studies | 16. Interpretation— a) Meaning: Give a cautious overall interpretation of results in the context of their limitations and in comparison with other studies b) Mechanism: Discuss underlying biological mechanisms that could drive a potential causal relationship between the investigated exposure and the outcome, and whether the gene-environment equivalence assumption is reasonable. Use causal language carefully, clarifying that IV estimates may provide causal effects only under certain assumptions c) Clinical relevance: Discuss whether the results have clinical or public policy relevance, and to what extent they inform effect sizes of possible interventions |  |
| Implications | 30 | Consider relevance to key groups (such as policy makers, service providers and service users). Consider implications for future research. | | 22. Implications—  - Discuss the implications of the overall results for clinical practice, policy, and science | 17. Generalizability—  Discuss the generalizability of the study results (a) to other populations, (b) across other exposure periods/timings, and (c) across other levels of exposure | - Did the authors investigate the potential for heterogeneity in causal effects?  - If YES, did the authors discuss heterogeneity of estimated causal effects and the possible impact on the generalizability of research findings? |
| Funding | 31 | Describe sources of funding and other support (such as supply of IPD), and the role in the systematic review of those providing such support. | | 23. Funding and role of sponsor—  - List all sources of funding or sponsorship for mediation analyses and the role of the funders/sponsors in the conduct of the study, writing of the manuscript, and decision to submit the manuscript for publication | 18. Funding— Describe sources of funding and the role of funders in the present study and, if applicable, sources of funding for the databases and original study or studies on which the present study is based | Source of funding |
|  |  |  | | 25. Data and Code—  - Authors are encouraged to provide a statement for sharing data and code for mediation analyses | 19. Data and data sharing—  Provide the data used to perform all analyses or report where and how the data can be accessed, and reference these sources in the article. Provide the statistical code needed to reproduce the results in the article, or report whether the code is publicly accessible and if so, where |  |
|  |  |  | | 24. Conflicts of interest and financial disclosures—  - State any ocnflicts of interest and financial disclosures for all authors | 20. Conflicts of interest—  All authors should declare all potential conflicts of interest |  |
